# Identifying characteristics and clinical conditions associated with hand grip strength in adults: the Project Baseline Health Study

**DOI:** 10.1038/s41598-024-55978-7

**Published:** 2024-04-18

**Authors:** Kenneth A. Taylor, Megan K. Carroll, Sarah A. Short, Adam P. Goode

**Affiliations:** 1 Department of Orthopaedic Surgery, Duke University School of Medicine, Durham, NC USA; 2 Duke Clinical Research Institute, Duke University School of Medicine, Durham, NC USA; 3 Verily Life Sciences, South San Francisco, CA USA; 4 Duke University School of Medicine, Population Health Sciences, Durham, NC USA

**Keywords:** Biomarkers, Medical research, Risk factors

## Abstract

Low hand grip strength (HGS) is associated with several conditions, but its value outside of the older adult population is unclear. We sought to identify the most salient factors associated with HGS from an extensive list of candidate variables while stratifying by age and sex. We used data from the initial visit from the Project Baseline Health Study (N = 2502) which captured detailed demographic, occupational, social, lifestyle, and clinical data. We applied MI-LASSO using group methods to determine variables most associated with HGS out of 175 candidate variables. We performed analyses separately for sex and age (< 65 vs. ≥ 65 years). Race was associated with HGS to varying degrees across groups. Osteoporosis and osteopenia were negatively associated with HGS in female study participants. Immune cell counts were negatively associated with HGS for male participants ≥ 65 (neutrophils) and female participants (≥ 65, monocytes; < 65, lymphocytes). Most findings were age and/or sex group-specific; few were common across all groups. Several of the variables associated with HGS in each group were novel, while others corroborate previous research. Our results support HGS as a useful indicator of a variety of clinical characteristics; however, its utility varies by age and sex.

## Introduction

Hand grip strength (HGS) is a noninvasive and reliably measured biomarker, most commonly assessed with a hydraulic hand dynamometer. HGS has been studied primarily in older adults, where it affords a consistent indicator of overall strength. HGS is most informative when paired with a measure of lower extremity strength^1^. In older adults, reduced HGS is associated with limitations in functional mobility and upper extremity activity^2–6^, and it represents a consistent indicator of disease status and mental health status. Lower HGS is associated with conditions such as prediabetes, diabetes (especially with neuropathy), and their clinical indicators (e.g., higher hemoglobin A1c; higher fasting blood glucose)^7–11^. It is also associated with multimorbidity^12–15^, malnutrition^16–19^, sleep impairment^20–23^, and psychological conditions such as depression^24–29^ and impaired cognition^30,31^.

Among healthy adults, HGS is highest in the 20–29 year age bracket. Healthy adults typically maintain their HGS through their 40s, with decreases thereafter; throughout, there are consistent differences by sex^32–41^. Some researchers have identified HGS cut points using age-appropriate reference values, intended to identify older individuals with sarcopenia or clinically relevant weakness, and to predict disease or mortality^42–47^. For example, older adults with HGS values below 16 kg for females or 26–27 kg for males, are likely to meet diagnostic criteria for sarcopenia and/or frailty and would benefit from further clinical assessment^35,42,48^. These cut points are generalizable to Japan, North America, Europe, and Australia, but not to other regions^49^.

HGS predicts future injury, disease, and mortality in older adults^50^. It is a strong predictor of incident cardiovascular disease, with estimated risk increasing 21% for each 5-kg decrease in HGS^51^, although it may not predict specific incident cardiovascular events^52^. Low HGS is associated with an increased risk of cardiovascular mortality^51,53,54^, with one study finding HGS to be a stronger predictor than systolic blood pressure for cardiovascular mortality and all-cause mortality^55^. Multiple cross-regional reports support the prognostic value of lower HGS as predictor of all-cause mortality^51,53,56–66^. Low HGS predicts future function in care-seeking and non-care-seeking populations^67–74^; it also predicts falls and osteoporotic fractures among older adults^75–82^, the latter likely being in direct relation to the associations that have been reported with physical strength, functional mobility, and bone mineral density.

Current evidence suggests HGS could be a biomarker with potential value for screening, diagnosis, and determining prognosis for a wide array of conditions. However, its value as an indicator or predictor of outcomes has not been well studied outside of the older adult population. Studies have typically investigated HGS alongside other variables as indicators or predictors of a specific outcome rather than comprehensively investigating clinical factors and conditions associated with HGS.

The Project Baseline Health Study (PBHS) is a prospective cohort study of U.S. adults that has collected a wide array of personal, behavioral, and clinical characteristics that together comprise data sufficiently rich to allow a deep physical and clinical characterization (phenotyping) of study participants^83,84^. In this analysis, we investigated the most salient factors associated with HGS by examining demographic, socioeconomic status-related health behaviors, medical conditions, symptoms, patient-reported outcomes, and laboratory measures in PBHS participants. We conducted these analyses stratifying by age and sex to account for expected differences between these groups.

## Methods

### Project Baseline Health Study (PBHS)

The PBHS (ClinicalTrials.gov identifier NCT03154346) is an ongoing longitudinal multicenter observational cohort study of U.S. adults. Participants contribute demographic, clinical, imaging, and laboratory data at annual visits. Detailed descriptions of study procedures and methods, including exclusion and inclusion criteria and institutional review board approval and enrollment procedures, have been published^83,84^.

In this study, we examined cross-sectional data collected at study participants’ baseline visit or collected remotely at that time via survey or connected device. Participants were enrolled between March 30, 2017 and April 26, 2019, and only data collected within 200 days of the enrollment visit were considered in the analysis. Participants were enrolled through sites affiliated with Stanford University (Palo Alto, CA), Duke University (Durham, NC and Kannapolis, NC), and California Health and Longevity Institute (CHLI; Los Angeles, CA).

### Hand grip strength

PBHS protocol recommended measuring HGS using the second handle position on the Jamar Plus + Hand Dynamometer (Patterson Medical). Participants were asked to perform 3 trials measuring peak grip force (kg) from a standing position with elbows by their side and arms flexed in front of them at a 90° angle. Study protocol recommended an approximate effort of 2–3 s of squeeze, with a 10–20 s break between each trial for dominant and non-dominant hands. If the difference between any 2 measures on a single hand was greater than 2.99 kg, a fourth trial was done after a 1-min resting period, and the minimum value from the four trials was dropped. For the purposes of this analysis, HGS was defined as the mean value from the 3–4 trials of the dominant hand. This approach is informed by the clinical assessment recommendations from the protocol of the American Society of Hand Therapists, although it has some differences (while the dynamometer handle is the same, participants took the test standing), and produces results comparable with other HGS measurement approaches^85,86^.

### Other variables

A detailed description of data collected from PBHS participants has been previously described^83^. We present a summary of the ascertainment and definition of the 175 variables eligible for entry into each model in the “Online Appendix”.

### Statistical analysis

Demographic and clinical characteristics including medical conditions, symptoms, patient-reported outcomes, and laboratory measures were described among participants who completed HGS assessments at their baseline visits. Male and female participants were described separately and they were classified into one of 3 cohort-based HGS percentile groups: lowest 25%, middle 50%, and highest 25%.

The Cochran–Armitage test for trend was used to test for associations between HGS and categorical variables of interest, the Spearman rank correlation test was used for continuous variables. Data collected within 200 days of participants’ baseline visits were considered nonmissing. Multiple imputation by chained equations (MICE) methods were used to address missing data^87^.

### LASSO regression

Least absolute shrinkage and selection operator procedures addressing multiply imputed data by using group methods^88^ (MI-LASSO) were employed to identify variables potentially associated with HGS, which was treated as a continuous outcome. Analyses were performed separately for male and female participants, further stratified by age (< 65 years vs. ≥ 65 years), resulting in 4 LASSO regression models. All variables were standardized to allow for meaningful comparison of coefficients within models. For each age-sex category, shrinkage of coefficients was controlled by a tuning parameter determined by the model that minimized the Bayesian Information Criterion.

### Linear regression

Linear regressions using variables selected from the LASSO were performed for each of the 4 age-sex categories to allow for more interpretable coefficient estimates for each variable identified by MI-LASSO. Regression estimates from each imputed dataset were pooled into a single value whose variance was then calculated using Rubin’s rules^89,90^.

Analyses were completed using R v3.6.3^91^. Data were imputed and linear regressions were performed using the MICE package v3.13.0^87^. LASSO regressions were carried out using the MI.LASSO function^88^. Missing data were imputed using observational data over a series of 5 instances, with 5 iterations per imputation. Figures were created with ggplot2 v3.3.0^92^.

## Results

Of the 2502 participants in the PBHS cohort, 2366 (95%) completed baseline HGS assessment; 1318 (55.7%) were female and 1048 (44.3%) were male. In this cohort, 63.5% of participants identified as White (64.2% of females; 62.6% of males), 16.1% as Black or African American (16.7%; 15.3%), 10.3% as Asian (8.3%; 12.8%), and 8.0% as Other (8.4%; 7.4%). The latter category included participants who identified as American Indian or Alaska Native, or Native Hawaiian or Other Pacific Islander, as well as those who indicated multiple racial identity backgrounds or provided a free-text response for race. Additionally, 11.4% of participants indicated Hispanic ethnicity (12.5%; 9.9%).

A plurality (41.8%) of the cohort attended study visits in Palo Alto, CA, while 21.6% attended in Kannapolis, NC; 19.7% attended in Durham, NC and 16.9% attended in Los Angeles, CA. Of the 1375 female participants included after imputation, 300 (21.8%) were ≥ 65 years of age; 282 (25%) of 1127 male participants were ≥ 65 years of age.

The 25th and 75th percentiles of HGS were 25 kg and 32 kg for female participants and 37 kg and 50 kg for male participants, respectively. Tables 1 and 2 describe associations between HGS and demographic data according to sex. Younger age was significantly associated with higher HGS in both male and female participants.

**Table 1:** Summary of demographic characteristics for female study participants across hand grip strength classifications.

Demographics	Female participants by grip strength			
	Lowest 25% (≤ 24 kg) (n = 352)	Middle 50% (> 24–32 kg) (n = 601)	Highest 25% (> 32 kg) (n = 365)	*P* value
Median age^a^, y (IQR)	61.6 (43.5–72.2)	50.9 (36.6–62.1)	43.0 (32.4–53.2)	< 0.001
Race, n (%)				
White	255 (72.4)	382 (63.6)	209 (57.3)	< 0.001
Black	33 (9.4)	88 (14.6)	99 (27.1)	< 0.001
Asian	30 (8.5)	57 (9.5)	22 (6.0)	0.22
Other	34 (9.7)	74 (12.3)	35 (9.6)	0.66
Native Hawaiian/Pacific Islander	4 (1.1)	8 (1.3)	2 (0.5)	
American Indian or Alaska Native	6 (1.7)	5 (0.8)	7 (1.9)	
Other Race with Free-Text Response	24 (6.8)	61 (10.1)	26 (7.1)	
Hispanic ethnicity, n (%)	50 (14.2)	75 (12.5)	40 (11.0)	0.19
Site, n (%)				
Durham, NC	44 (12.5)	112 (18.6)	122 (33.4)	< 0.001
Kannapolis, NC	112 (31.8)	119 (19.8)	64 (17.5)	< 0.001
Los Angeles, CA	39 (11.1)	128 (21.3)	62 (17.0)	0.04
Palo Alto, CA	157 (44.6)	242 (40.3)	117 (32.1)	0.06

*P* values calculated using Spearman correlation or Cochran–Armitage tests.

^a^Pearson’s product-moment correlation = − 0.35.

**Table 2:** Summary of demographic characteristics for male study participants across hand grip strength classifications.

Demographics	Male participants by grip strength			
	Lowest 25% (≤ 37 kg) (n = 244)	Middle 50% (> 37–50 kg) (n = 534)	Highest 25% (> 50 kg) (n = 270)	*P* value
Median age^a^, y (IQR)	66.0 (47.1–74.4)	51.2 (34.3–64.7)	41.4 (31.1–50.7)	< 0.001
Race, n (%)				
White	160 (65.6)	349 (65.4)	147 (54.4)	0.008
Black	27 (11.1)	76 (14.2)	57 (21.1)	0.001
Asian	41 (16.8)	63 (11.8)	30 (11.1)	0.06
Other	16 (6.6)	46 (8.6)	36 (13.3)	0.008
Native Hawaiian/Pacific Islander	2 (0.8)	4 (0.7)	5 (1.9)	
American Indian or Alaska Native	0 (0)	5 (0.9)	4 (1.5)	
Other Race with Free-Text Response	14 (5.7)	37 (6.9)	27 (10.0)	
Hispanic ethnicity, n (%)	21 (8.6)	55 (10.3)	28 (10.4)	0.51
Site, n (%)				
Durham, NC	28 (11.5)	99 (18.5)	61 (22.6)	0.001
Kannapolis, NC	64 (26.2)	95 (17.8)	57 (21.1)	0.18
Los Angeles, CA	19 (7.8)	100 (18.7)	53 (19.6)	< 0.001
Palo Alto, CA	133 (54.5)	240 (44.9)	99 (36.7)	< 0.001

*P* values calculated using Spearman correlation or Cochran–Armitage tests.

^a^Pearson’s product-moment correlation = − 0.37.

### Female participants

**Figure 1:**
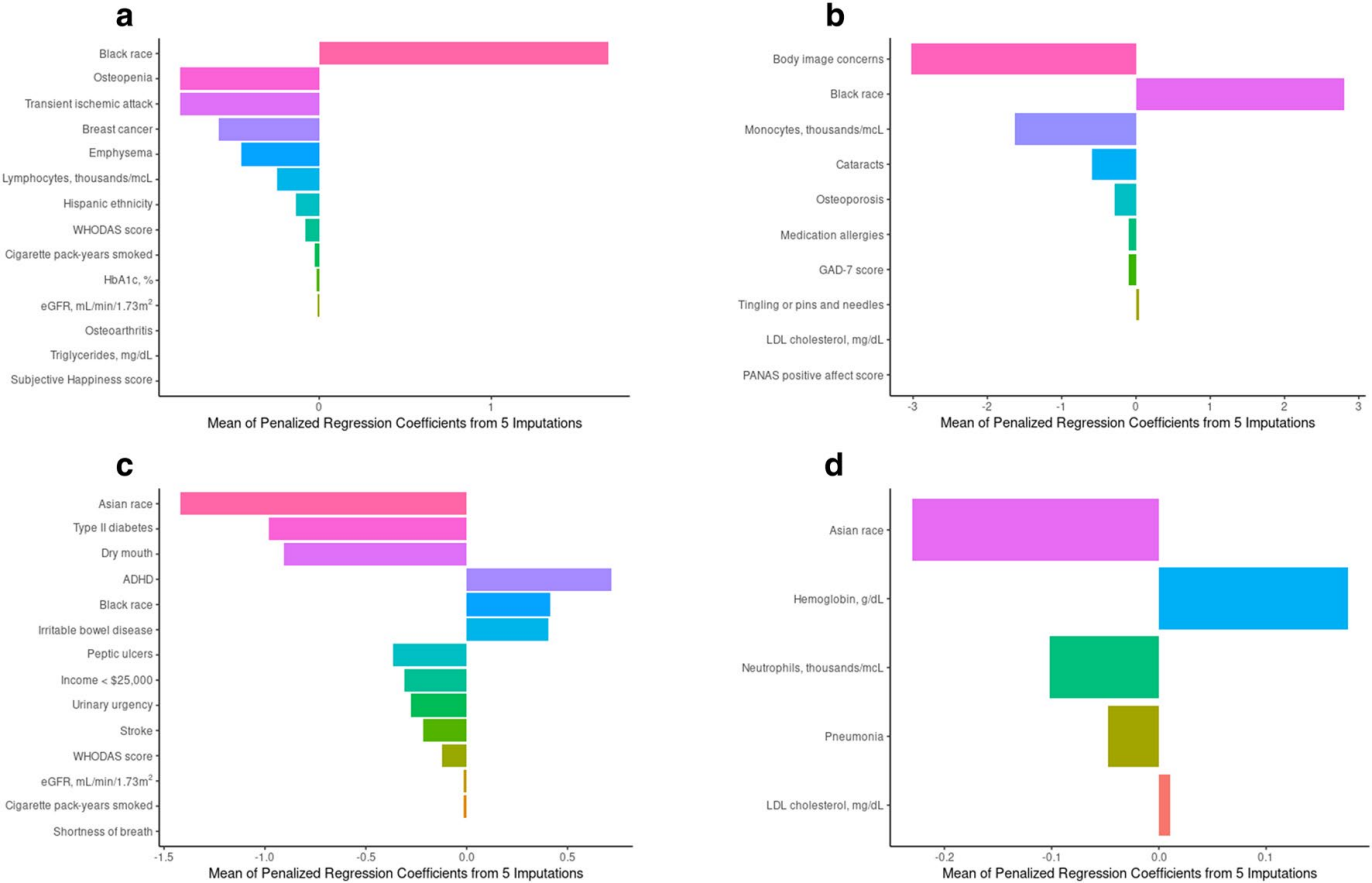
(**a**) Characteristics associated with hand grip strength after MI-LASSO regression using multiply imputed data: females study participants aged < 65 years (N = 1075). Abbreviations: WHODAS, World Health Organization Disability Assessment Schedule 2.0; HbA1c, hemoglobin A1c; eGFR, estimated glomerular filtration rate. (**b**) Characteristics Associated with hand grip strength after MI-LASSO regression using multiply imputed data: females study participants ≥ 65 years (N = 300). Because no study participants in this age/sex group had type 1 diabetes mellitus, this variable was removed from the model for this group. Abbreviations: GAD-7, Generalized Anxiety Disorder; LDL, low density lipoprotein; PANAS, Positive and Negative Affect Schedule. (**c**) Characteristics associated with hand grip strength after MI-LASSO regression using multiply imputed data: male study participants aged < 65 years (N = 845). Abbreviations: ADHD, attention-deficit/hyperactivity disorder; WHODAS, World Health Organization Disability Assessment Schedule 2.0, eGFR, estimated glomerular filtration rate. (**d**) Characteristics associated with hand grip strength after MI-LASSO regression using multiply imputed data: male study participants aged ≥ 65 years (N = 282). Because no study participants in this age/sex group had fibromyalgia, this variable was removed from the model for this group. Abbreviations: LDL, low-density lipoprotein.

We observed an association between Black race and higher HGS in all female participants, regardless of age (Fig. 1a,b). In regression models using variables selected from the MI-LASSO, (Table 3) Black race was associated with approximately 3–4 kg higher HGS in female participants regardless of age group. No other variables identified by MI-LASSO were shared across age groups among females.

**Table 3:** Estimates (95% CI) from linear regression models using covariates selected in MI-LASSO regressions.

Variable	Female, < 65 years	Female, ≥ 65 years	Male, < 65 years	Male, ≥ 65 years
(Intercept)	35.4 (31.9, 38.9)	22.9 (18.1, 27.8)	53.0 (50.0, 56.0)	24.4 (12.1, 36.7)
Black race	3.16 (2.12, 4.21)	4.27 (2.25, 6.28)	3.04 (1.26, 4.82)	NA
Hispanic ethnicity	− 1.05 (− 2.20, 0.11)	NA	NA	NA
Cigarette pack-years smoked	− 0.05 (− 0.10, − 0.01)	NA	− 0.04 (− 0.10, 0.02)	NA
Breast cancer	− 3.7 (− 6.53, − 0.86)	NA	NA	NA
COPD	− 1.83 (− 4.61, 0.96)	NA	NA	NA
Osteoarthritis	− 1.33 (− 2.52, − 0.14)	NA	NA	NA
Osteopenia	− 4.09 (− 6.49, − 1.70)	NA	NA	NA
Transient ischemic attack	− 7.02 (− 12.1, − 1.94)	NA	NA	NA
Subjective Happiness score	0.07 (− 0.03, 0.16)	NA	NA	NA
WHODAS score	− 0.09 (− 0.17, − 0.01)	NA	− 0.24 (− 0.40, − 0.09)	NA
Triglycerides, per 10 mg/dL increase	− 0.01 (− 0.07, 0.05)	NA	NA	NA
HbA1c, %	− 0.28 (− 0.70, 0.15)	NA	NA	NA
Lymphocytes, per 1000/mcL increase	− 0.71 (− 1.45, 0.02)	NA	NA	NA
eGFR, mL/min/1.73m^2^	− 0.04 (− 0.06, − 0.02)	NA	− 0.06 (− 0.09, − 0.03)	NA
Cataracts	NA	− 1.42 (− 2.65, − 0.20)	NA	NA
Osteoporosis	NA	− 1.51 (− 3.08, 0.07)	NA	NA
Body image concerns	NA	− 5.52 (− 8.89, − 2.15)	NA	NA
Tingling or pins and needles	NA	1.35 (− 0.47, 3.16)	NA	NA
Medication allergy	NA	− 0.95 (− 2.13, 0.22)	NA	NA
PANAS positive affect score	NA	0.06 (− 0.05, 0.17)	NA	NA
GAD-7 score	NA	− 0.20 (− 0.37, − 0.02)	NA	NA
LDL cholesterol, per 10 mg/dL increase	NA	0.17 (0.01, 0.33)	NA	0.33 (0.03, 0.63)
Monocytes, per 1000/mcL increase	NA	− 3.83 (− 8.09, 0.43)	NA	NA
Income < $25,000	NA	NA	− 1.56 (− 3.98, 0.86)	NA
Asian race	NA	NA	− 3.00 (− 4.80, − 1.19)	− 4.88 (− 8.85, − 0.92)
ADHD	NA	NA	3.50 (1.01, 6.00)	NA
Type 2 diabetes	NA	NA	− 2.41 (− 4.58, − 0.25)	NA
Irritable bowel disease	NA	NA	4.77 (0.94, 8.60)	NA
Peptic ulcers	NA	NA	− 4.81 (− 9.99, 0.37)	NA
Stroke	NA	NA	− 7.00 (− 15.2, 1.19)	NA
Dry mouth	NA	NA	− 3.08 (− 5.87, − 0.30)	NA
Shortness of breath	NA	NA	− 1.22 (− 4.25, 1.81)	NA
Urinary urgency	NA	NA	− 2.81 (− 6.29, 0.68)	NA
Pneumonia	NA	NA	NA	− 3.59 (− 6.77, − 0.41)
Hemoglobin, g/dL	NA	NA	NA	0.98 (0.15, 1.81)
Neutrophils, thousands/mcL	NA	NA	NA	− 0.87 (− 1.60, − 0.13)

Variance estimates were calculated using Rubin’s rules.

ADHD, attention deficit/hyperactivity disorder; COPD, chronic obstructive pulmonary disorder; eGFR, estimated glomerular filtration rate; GAD, generalized anxiety disorder; HbA1c, hemoglobin A1c (glycated hemoglobin); LDL, low-density lipoprotein; PANAS, positive and negative affect schedule; WHODAS, World Health Organization Disability Assessment Schedule.

We identified several variables associated with HGS that were specific to age group. In younger female participants, there were several categorical variables associated with ≥ 2 kg lower HGS: history of breast cancer, osteopenia, and transient ischemic attack (TIA). Also in younger female participants, some continuous variables had an inverse association with HGS (increasing values associated with lower HGS), including estimated glomerular filtration rate (eGFR), World Health Organization (WHO) Disability Assessment Schedule (WHODAS 2.0) total score, and cigarette pack-years smoked. In older female participants, reporting body image concerns was associated with ≥ 2 kg lower HGS. Continuous variables where increasing values were associated with lower HGS included low-density-lipoprotein (LDL) cholesterol, Generalized Anxiety Disorder-7 (GAD-7) score, and absolute monocyte count.

### Male participants

Among male participants (Fig. 1c,d; Table 3), Asian race was associated with approximately 3–5 kg lower HGS.

In younger male participants, Black race was associated with approximately 3 kg higher HGS. In addition, there were categorical variables associated with ≥ 2 kg lower HGS, including type 2 diabetes mellitus (T2DM), dry mouth, peptic ulcers, and stroke. Conversely, attention-deficit/hyperactivity disorder (ADHD) and irritable bowel disease were associated with ≥ 2 kg higher HGS. Also in younger male participants, some continuous variables had an inverse association (increasing values associated with lower HGS), including eGFR and WHODAS 2.0 score.

In older male participants, pneumonia was associated with ≥ 2 kg lower HGS. Additionally, higher hemoglobin and LDL cholesterol levels were associated with higher HGS while higher neutrophil count was associated with lower HGS.

## Discussion

In this study and as a means of high-throughput hypothesis generation, we sought to identify the most salient factors associated with HGS out of a wide range of clinical variables when stratifying by both sex and age. We used a deeply phenotyped cohort, meaning, a cohort where an intensive effort for data collection had taken place, including clinico-physiologic tests, as well measurements of general function and health status^83^. Our study is the first to stratify participants by age in order to investigate variables associated with HGS, allowing comparisons across both sex and age. Our results underscore previous findings that HGS is associated with several health dimensions of interest and that these associations vary by age and sex. In addition to confirming a number of previously reported associations, our analyses also identified several novel associations that warrant further investigation in specific age and sex groups. Novel associations include eGFR in individuals aged < 65, LDL cholesterol in individuals aged ≥ 65, psychosocial factors such as body image concerns, subjective happiness, and PANAS positive affect score in females and ADHD in males aged < 65, and immune cells in males aged ≥ 65 and females overall.

We found a consistent association of race and HGS across sex and age categories to varying degrees, findings that align with published literature reporting differences in HGS across race categories^2,32,93,94^. These observations may be explained at least in part by differences in body composition often seen across ethnic or racial backgrounds^95,96^; however, the ultimate reasons for these differences are likely complex, as social factors and experiences associated with race such as health and wealth disparities, impacts of racism, and other unmeasured racialized experiences will be jointly captured by the race variable in our regression models^97^.

The only other factor commonly associated with HGS across more than 2 stratified groups was immune cell count; however, the specific type of white blood cell associated with HGS differed between groups. An association between higher absolute monocyte count and lower HGS was observed in female participants aged ≥ 65 years, while an association between higher total lymphocyte count and lower HGS was seen in female participants aged < 65 years. Among male participants aged ≥ 65 years, higher total neutrophil count was also associated with lower HGS, but we observed no associations of immune cell-based measures with HGS among younger male participants. Few studies have reported on associations between HGS and immune cells, although there is one report of an association between neutrophil dysfunction and age exaggerated by frailty (for which HGS is an indicator) in older adults^98^. Additionally, only one study in older adults has reported on the association between HGS and heat shock protein expression in monocytes and lymphocytes^99^. In our study, we found absolute monocyte count to be associated with the largest estimated decrease in predicted HGS, with each 1000 mcL increase in absolute monocyte count being associated with a nearly 4-kg lower HGS. In the other groups, immune cells other than absolute monocyte count were associated with HGS to a lesser degree, with the estimated HGS being < 1 kg lower per 1-unit increase.

In addition to the associations between race and HGS, female participants across age groups in our study also showed associations between bone mineral density conditions and HGS. Previous studies have reported an association between low HGS in older adults (primarily perimenopausal and postmenopausal women) and insufficient bone mineral density at sites remote from the upper extremity (e.g., spine and hip)^100–108^. HGS has also been identified as a viable supplemental predictor of incident fractures when traditional bone mineral density scans (i.e., dual-energy X-ray absorptiometry) are unavailable or cost-prohibitive^77–82^. Our results, which show that lower HGS is associated with osteoporosis in older female participants and with osteopenia in female participants aged < 65 years, provide additional support for these previous reports. We did not observe associations between HGS and prevalent osteopenia or osteoporosis in male participants, but others have reported this association in a large, nationally representative sample when limiting their analysis to adults aged ≥ 40 years^109^.

Other than race and bone mineral density conditions, no associations with HGS were common across age groups among female participants. In female participants aged < 65, several of the diagnoses associated with lower HGS are associated with worse overall physical function and strength: breast cancer, TIA, COPD, and osteoarthritis.

Younger female participants showed a small association between higher subjective happiness scores and higher HGS; however, there were multiple indicators of mental health associated with HGS in female participants aged ≥ 65. Most notable among these were body image concerns and GAD-7 score, both of which were associated with lower HGS. A recent report from the Korea National Health and Nutrition Examination Survey has also found an association between body image concerns and lower HGS in older adults. In this study, the authors reported that HGS was associated with accuracy of weight estimation and behaviors related to weight loss or maintenance in both male and female participants aged ≥ 60 years, but not among younger study participants^110^.

A number of studies have investigated associations between depression and HGS; however, only 2 have reported associations between prevalent GAD and HGS. The first, an Irish study in adults aged ≥ 50 years, found an association between prevalent GAD and HGS in both female and male participants^111^. The second study reported lower HGS in female participants with GAD compared with male participants, especially with those with age of onset ≥ 40 years^112^.

We observed no common factors associated with HGS across age categories other than race, but each age group did have different associations not previously reported. In male participants aged ≥ 65, higher hemoglobin levels were associated with higher HGS, while a history of pneumonia was associated with lower HGS. In male participants aged < 65 years, a history of ADHD or irritable bowel disease was associated with higher HGS. Although the association of ADHD with multiple domains of upper limb function has been previously demonstrated in children^113^, our study is the first to report an association between ADHD and HGS in adults. Similar to younger female participants, some of the diagnoses associated with lower HGS in younger male participants are generally associated with worse physical function and overall strength, such as T2DM and stroke.

Symptoms unexpectedly associated with lower HGS were dry mouth (HGS approximately 3 kg lower) and peptic ulcers (approximately 5 kg lower). To our knowledge, associations between these variables and HGS have not been previously reported. However, we note that the association between HGS and dry mouth may be due at least in part to medication data not being available for inclusion in our analyses. This younger male group was also the only group overall to also have a socioeconomic status indicator associated with HGS, with income < $25,000 associated with lower HGS.

Overall, more variables were associated with HGS in participants aged < 65 years regardless of sex. In this age group, higher WHODAS 2.0 score, pack-years of smoking, and eGFR were associated with lower HGS. The WHODAS 2.0, a standardized generic measure of health and disability, comprises 6 domains relating to cognition, mobility, self-care, getting along, life activities, and participation^114^. Our finding that a higher total WHODAS 2.0 score (i.e., worse disability) was associated with lower HGS is not unexpected, given previously reported associations between HGS and disability domains either directly (e.g., cognition, mobility, physical function, activity)^2–6,30,31,67,68^ or indirectly (e.g., symptoms associated with depression)^24–29^ addressed by WHODAS 2.0. However, studies linking WHODAS 2.0 disability domains (using alternative metrics) and HGS were done primarily in older adult cohorts, and we did not find an association of WHODAS 2.0 score with HGS for those aged ≥ 65 years. We found such association in both female and male participants < 65, and the decrease in estimated HGS per unit increase (worse function) in WHODAS 2.0 was more than double for male participants.

Smoking status has been previously associated with HGS and other measures of physical function in older adults, an association not observed in this study^115,116^. A study of a cohort of British participants aged < 65 showed an association between pack-years of smoking and several physical function measures, but not HGS^117^. Of note, in our study this association was relatively weak, requiring a 20-pack-year smoking history to be associated with an approximate 1-kg reduction in HGS.

Higher eGFR was also associated with lower HGS in participants aged < 65 years but was not associated in participants ≥ 65. These findings are consistent with previous studies showing that eGFR, when calculated according to the method used in this study, is not associated with HGS in older adults^118,119^. However, eGFR calculated by alternative methods has been associated with HGS in older adults in at least one other study^120^. Findings in our younger cohort indicate that higher eGFR (better kidney function) is associated with lower HGS. This apparent paradox may, like similar paradoxes^121,122^, be due to collider bias, which can be induced when selection into the study is affected by both the variable of interest (eGFR) and the outcome (HGS), or when some other third variable affected by these two variables is adjusted for in statistical analysis^123,124^. To our knowledge, no other studies have examined the association between eGFR and HGS in participants aged < 65 years.

Among participants aged ≥ 65 years, higher LDL cholesterol level was associated with higher HGS, regardless of sex. Although this association was present for both older female and male participant groups, the estimated increase in HGS per 10 mg/dL increase in LDL was twice as high in males. Previous reports from other countries have shown consistent associations between LDL and HGS in older adults^125,126^. Previous studies have also linked HGS with cardiovascular disease and mortality^51,53–55^ and HGS with other indicators of cardiometabolic health/risk (e.g., high-density-lipoprotein cholesterol; blood pressure)^125,127^. However, LDL was the only cardiometabolic risk factor in our results associated with HGS in older adults of either sex.

We note a number of limitations to our study. First, we used cross-sectional data and methods focused on identifying factors most strongly associated with HGS as a means of high-throughput hypothesis generation rather than attempting to estimate the causal effect of any single specific variable on HGS^128^. Establishing causality would necessitate an alternative approach. Some of the associations described (e.g., eGFR, LDL) may seem paradoxical if interpreted under the assumption that they reflect a causal mechanism. Therefore, readers are cautioned against interpreting our results as estimates of any causal effect. Second, our study sample was limited to populations in North Carolina (Durham and Kannapolis) and California (Los Angeles, Palo Alto). The sample comprised adults older than the national average and had less Hispanic representation (11.4%) than the overall U.S. population (18.5%)^129^. Because of this, the generalizability of our findings to the overall U.S. adult population may be limited.

This study identified several novel salient factors associated with HGS when stratifying by sex and age (< 65 vs. ≥ 65 years) and observed associations that confirm previously published work. Some of these associations were common across age groups or sex groups, but several factors associated with HGS were specific to both age and sex. These results support the hypothesis that HGS may be a useful indicator of a variety of prevalent conditions, as it was associated with a range of clinical factors, complaints, and laboratory blood measures. The novel associations we have identified may merit further investigation. If confirmed, then HGS may also be a useful health indicator that could be incorporated into general assessments to enrich the understanding of a patient’s condition across a variety of domains. Further research would be warranted to estimate the direction and magnitude of any causal relationships between HGS and novel factors where biologically plausible.

### Supplementary Information

Supplementary Information.

## Data Availability

The de-identified Project Baseline Health Study (PBHS) data corresponding to this study are available upon request for the purpose of examining its reproducibility. Interested investigators should direct requests to sarahshort@verily.com. Requests are subject to approval by PBHS governance.
